# Greater frequency of CD5-negative CD8^+^ T cells against human immunodeficiency virus type 1 than other viruses is consistent with adaptation to antigenic variation

**DOI:** 10.1186/1742-6405-11-30

**Published:** 2014-09-15

**Authors:** Stephen J Penney, Maureen E Gallant, Michael D Grant

**Affiliations:** 1 Immunology and Infectious Diseases Program, Division of BioMedical Sciences, Faculty of Medicine, Memorial University of Newfoundland, St. John’s, NL A1B 3 V6, Canada; 2 H1803-Immunology, Faculty of Medicine, Memorial University of Newfoundland, 300 Prince Philip Drive, St. John’s, NL A1B 3 V6, Canada

**Keywords:** CD5, CD8^+^ T cell, HIV, T cell receptor, Peptide, Avidity, HLA class I

## Abstract

**Background:**

The CD5 protein antagonizes phosphorylation events downstream of T cell receptor (TCR) engagement to decrease T cell responsiveness. CD5-negative T cell clones respond preferentially over their CD5^+^ counterparts against cells with low human histocompatibility-linked leukocyte antigen (HLA) levels. In human immunodeficiency virus type 1 (HIV-1) infection, CD5^-^CD8^+^ T cells increase in prevalence with disease progression.

**Methods:**

To investigate potential causes of this expansion of CD5^-^CD8^+^ T cells in HIV-1 infection, we compared CD5 expression on CD8^+^ T cells reactive against HIV-1 peptides, common viral peptides and a self peptide that together span a broad range of TCR avidities in the context of the common HLA-A2 class I restriction molecule. Following stimulation, CD5 expression on peptide-specific CD8^+^ T cells was assessed by flow cytometry.

**Results:**

In healthy controls, there was no significant difference in the CD5^+^ percentage of CD8^+^ T cells specific for common viral peptides, but a lower percentage of those responding against a common self peptide expressed CD5. The same relationship occurred in HIV-infected individuals, however, a lower percentage of HIV peptide-specific CD8^+^ T cells than other viral peptide-specific CD8^+^ T cells expressed CD5. In terms of overall CD5 expression level at the peptide-specific responder population level, HIV-specific CD8^+^ T cells resembled those responsive against the self peptide, despite much higher avidity TCR/HLA/peptide interactions.

**Conclusions:**

This deficit in CD5 expression selective for HIV-specific CD8^+^ T cells is consistent with *in vivo* adaptation to low avidity HIV peptide variants and has potential consequences for CD8^+^ T cell expansion, cross-reactivity and autoreactivity.

## Background

Selective recognition of peptides within particular major histocompatibility complex (MHC) molecules by clonotypic T cell receptors (TCR) underlies both the specificity and mechanics of T cell activation. While the overall strength or avidity of the cognate TCR/peptide/MHC interaction must itself exceed a certain threshold, non-polymorphic accessory, co-receptor and other molecules can modulate the tenor of signal transduction and sway the outcome of antigen-specific recognition. The group B cysteine-rich scavenger receptor CD5 modulates antigen-specific signaling on virtually all T cells in a ligand-independent manner [1-5]. When T cells form conjugates with antigen-presenting cells, CD5 colocalizes with TCRs engaging cognate MHC/peptide complexes within the immunological synapse and recruits SHP phosphatases to the area through its cytoplasmic tyrosine inhibitory motif (ITIM), [3,6]. Thus, localized antagonism of phosphorylation events by CD5 diminishes TCR-derived activation signals [7]. With higher levels of CD5, the interaction between TCR and MHC/peptide complex must be stronger to overcome such inhibition. This implies a relationship where the level of CD5 expression on a T cell could fine tune the avidity of cognate TCR/MHC/peptide complex interactions required for T cell activation [8].

The first direct evidence that CD5 represses T cell activation was hyperresponsiveness of thymocytes from CD5-knockout mice to TCR signaling, implicating thymic selection in tuning CD5 expression levels to TCR avidity [4,9]. As thymocytes mature, CD5 and TCR levels increase in parallel, which should facilitate early positive selection of CD5^low^ thymocytes by lower avidity interactions and hinder late negative selection of CD5^high^ thymocytes by higher avidity interactions [10]. The modulating effect of CD5 during thymic selection and tuning of CD5 levels potentially extends the overall avidity range of the selected TCR repertoire, especially at the higher end. In TCR transgenic mice, autoreactive T cells with high levels of CD5 expression escape negative thymic selection, but peripheral downregulation of CD5 releases their autoreactivity [11-13]. There is also a striking difference in vitro in the behaviour of T cell clones derived from tumour-infiltrating lymphocytes in that only CD5-negative clones are activated by tumour cells expressing low levels of cognate MHC/peptide complexes [14]. Since the clones have identical TCR, CD5 expression is not inexorably hard-wired to TCR avidity and under certain conditions, adapts to the antigenic environment or to homeostatic pressures to either increase or decrease T cell responsiveness [15-17]. However, the role that CD5 expression plays in shaping peripheral T cell responses against foreign antigens in non-transgenic animals is completely obscure.

Substantial increases in CD5-negative T cells were reported in human immunodeficiency virus type 1 (HIV-1) infection, especially in its later stages [18]. These data predate the introduction of highly active antiretroviral therapy (HAART), suggesting a relationship between robust HIV replication and selective expansion of CD5-negative T cells. The CD5-negative T cells observed were predominantly CD8^+^, but neither their specificity nor function was investigated [18]. Subsequent studies of CD8^+^ T cells in HIV infection have reported abnormal promiscuity amongst HIV peptide-specific CD8^+^ T cells, including unexpected cross-reactivity against multiple HIV peptides, other viral peptides and self peptides [19-21]. The high mutation rate of HIV generates multiple variants of the peptide epitopes activating HIV-specific CD8^+^ T cells. While some are escape mutants, many variants will bind the same class I MHC protein, but mediate higher or lower avidity interactions with the TCR of CD8^+^ T cells initially activated by the wild type peptide. In this setting, downregulation of CD5 could compensate for loss of avidity, selectively reduce the percentage of HIV-specific CD8^+^ T cells expressing CD5 and increase the likelihood of CD8^+^ T cell cross-reactivity and autoreactivity.

To investigate the possibility of selective reduction in the percentage of HIV peptide-specific CD8^+^ T cells expressing CD5, we compared CD8^+^ T cells specific for HIV peptides, non-HIV viral peptides, and a low avidity self peptide in HIV-infected individuals and healthy controls. Our rationale was that CD8^+^ T cells reactive against peptides from viruses with much less variation than HIV would not undergo selection for CD5-downregulation and a higher percentage would express CD5. Dominant CD8^+^ T cell epitopes from common influenza (flu), cytomegalovirus (CMV) and Epstein-Barr virus (EBV) viruses were selected for comparison with dominant CD8^+^ T cell epitopes from HIV and the common self peptide. We chose these particular viruses and epitopes restricted to HLA-A2 because widespread exposure of the general population to flu, CMV and EBV and the high frequency of HLA-A2 in the population make the comparison feasible with less subject screening. Healthy controls expressing HLA-A2 were initially screened by interferon-gamma (IFN-γ) ELIspot assay for reactivity against the common viral peptides and self peptide, while HIV-infected individuals expressing HLA-A2 were screened against the common viral peptides, HIV peptides and common self peptide. For those individuals reactive by ELIspot, we followed up with CD8^+^ T cell proliferation assays, which allowed us to assess by flow cytometry the percentage of CD8^+^ T cells expressing CD5 in the population responding to the peptide. The percentage of CD8^+^ T cells expressing CD5 varies with their specificity and with the underlying avidity of TCR/peptide/MHC interactions.

## Results

### Percentage of circulating CD8^+^ T cells expressing CD5 in HIV-infected and uninfected individuals

**Figure 1:**
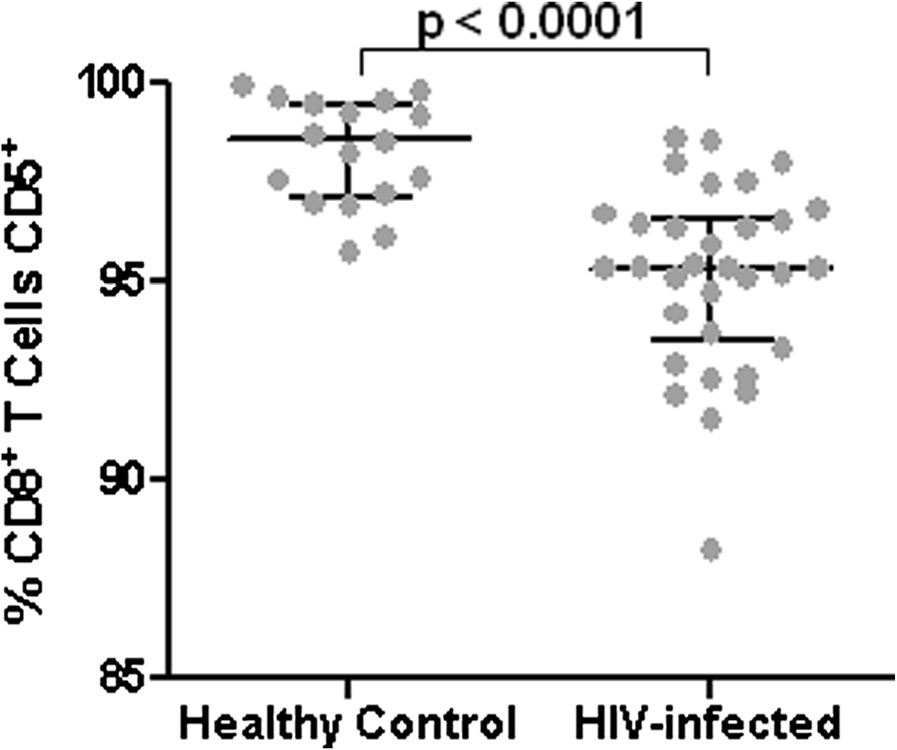
**Comparison of the percentage of circulating CD8**^**+ **^**T cells expressing CD5 in HIV-infected individuals and healthy controls.** The median percentage of circulating CD8^+^ T cells expressing CD5 measured by flow cytometry is shown for each group by a horizontal line bisecting the data points with upper and lower horizontal lines in each group indicating interquartile range. Significant difference between the medians is indicated by the p value above the line spanning the data sets (Mann–Whitney test).

Before assessing the percentages of peptide-specific CD8^+^ T cells expressing CD5 in HIV-infected individuals and healthy controls, we measured the percentage of CD8^+^ T cells expressing CD5 in the general CD8^+^ T cell populations of 32 HIV-infected individuals and 17 healthy controls by flow cytometry. The median percentage of CD8^+^ T cells expressing CD5 was significantly lower for the HIV-infected individuals (median = 95.3, interquartile range 93.5-96.6 versus median 98.6, interquartile range 97.1-99.5, p < 0.0001, Mann Whitney test). While HIV-infection significantly reduces the proportion of CD8^+^ T cells expressing CD5, a median of > 95% of all CD8^+^ T cells expressed CD5 in both groups (Figure 1). Thus, in the post-HAART era, with HIV replication effectively suppressed, a small subset of CD8^+^ T cells is selectively affected by HIV infection in terms of CD5 expression. Subsequent experiments addressed the potential contribution of different CD8^+^ T cell specificities to this effect.

### Percentage of CMV, EBV, flu and IP-30 self peptide-specific CD8^+^ T cells expressing CD5 in healthy controls

**Figure 2:**
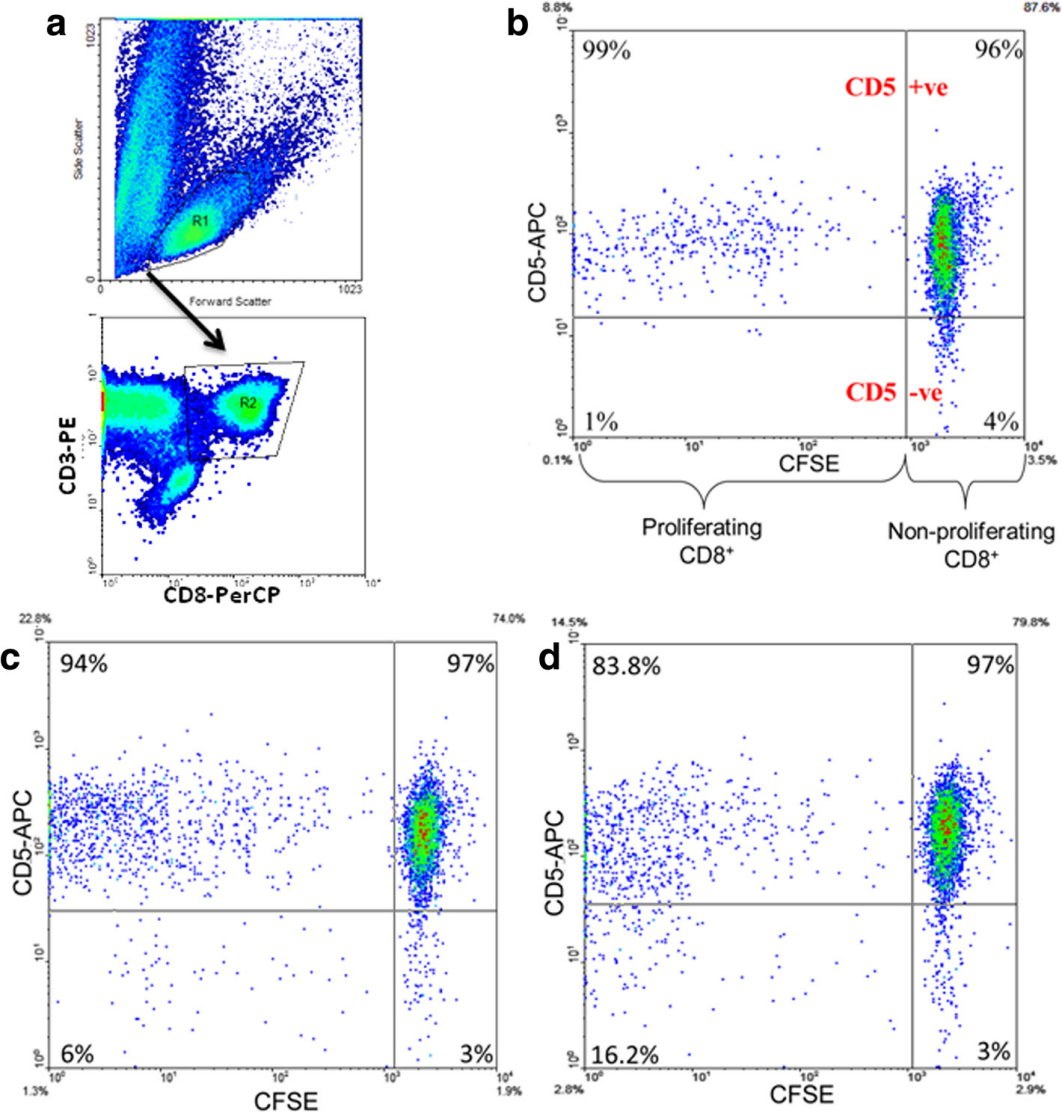
**Assessment of the percentage of peptide-specific CD8**^**+ **^**T cells expressing CD5.** Responder cells were labeled with CFSE, stimulated with the desired peptides for 7 days, surface stained for CD3, CD8 and CD5 and then analyzed by flow cytometry. CD3^+^CD8^+^ lymphocytes were positively gated from the live lymphocyte population **(a)** and the fraction of proliferating cells expressing CD5 was calculated by visualizing CFSE fluorescence intensity versus CD5 in plots **b**-**d**. Proliferating, peptide responsive cells have lower CFSE fluorescence intensity than the parental non-responding, non proliferating population **(b)**. Numbers in the quadrants of plots **(b-d)** represent CD5^+^ (upper quadrants) and CD5-negative (lower quadrants) percentages of the non-responding (right hand quadrants) and peptide responsive CD8^+^ T cell populations (left hand quadrants). Representative examples where the percentage of peptide-responsive, proliferating cells not expressing CD5 spans a range of 1% **(b)** to 16% **(d)** are shown for CD8^+^ T cell responses to the CMV **(b)** PR **(c)** and Gag **(d)** peptides described in Table 1.

**Figure 3:**
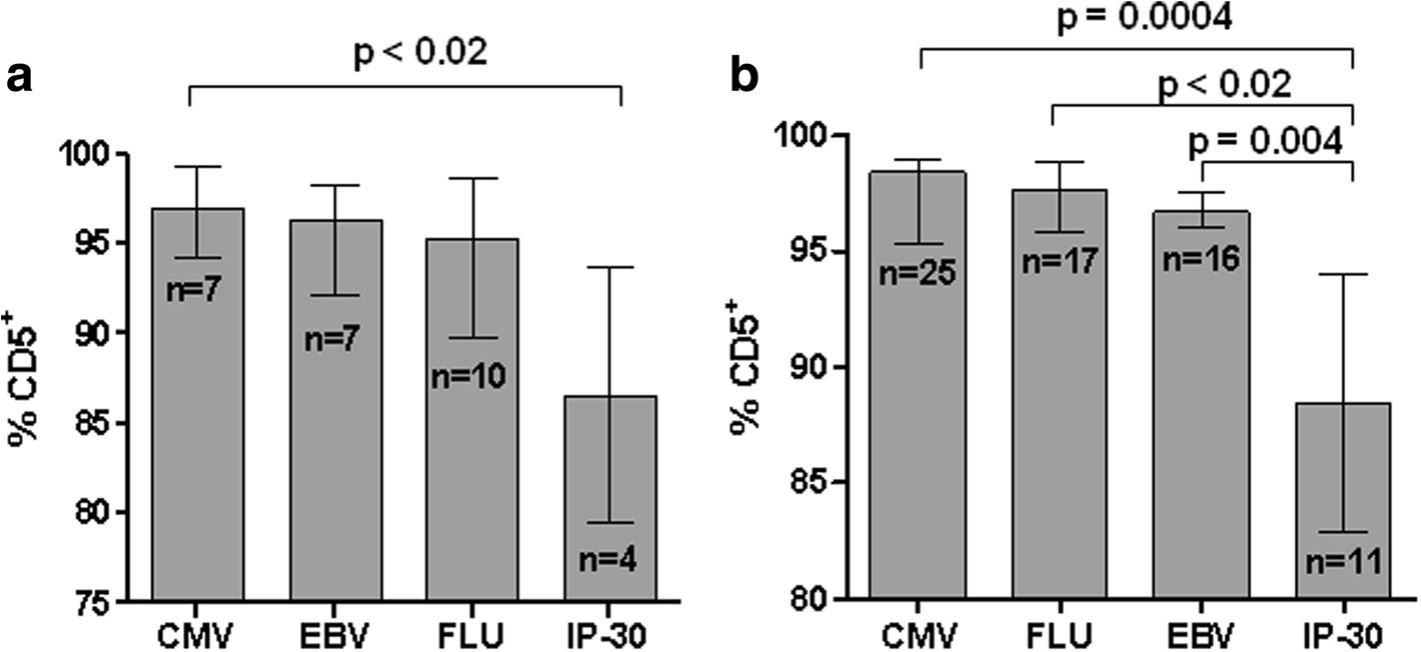
**Comparison of the percentage of non-HIV peptide-specific CD8**^**+ **^**T cells expressing CD5 for healthy controls and HIV-infected individuals.** Solid columns represent median values of the percentage of peptide-specific CD8^+^ T cells expressing CD5 for each individual peptide (identified below the column) with error bars showing interquartile range for healthy controls **(a)** and HIV-infected individuals **(b)**. The number of individuals in each group with specific proliferation against each individual peptide is indicated by the n value within the column. Significant differences between medians are shown by p values above lines bracketing the relevant groups (Mann–Whitney test).

We first analyzed CD8^+^ T cells proliferating in response to the common non-HIV viral peptides and IP-30 self-peptide in HLA-A2^+^ healthy controls to test if a general relationship between avidity of peptide/TCR interactions and the percentage of responding CD8^+^ T cells expressing CD5 was apparent. Avidities of the non HIV and HIV peptides used in this study are listed in Table 1. The percentage of peptide-specific CD8^+^ T cells expressing CD5 was estimated by flow cytometry after gating on CD8^+^CD3^+^ cells and visualizing CD5 expression on CFSE^low^ cells as shown for representative examples in Figure 2. For the 3 foreign peptides, medians of 97.0%, 96.3% and 95.3% of CMV, EBV and flu peptide-specific CD8^+^ T cells respectively, expressed CD5, with no significant difference between medians (Figure 3a). Only 4 of 24 uninfected controls tested had CD8^+^ T cell proliferation stimulated by the low avidity IP-30 self peptide, with a median of 86.5% of the responding CD8^+^ T cells expressing CD5. While the non-HIV viral peptides tested varied from 1.0 to 500 nM in TCR avidity, the avidity difference over this range was not reflected in significantly different percentages of responding CD8^+^ T cells expressing CD5. However, a significantly lower percentage of CD8^+^ T cells responding against the IP-30 self peptide (avidity = 2.0 μM) expressed CD5 compared to the CD8^+^ T cells responding against the highest avidity CMV peptide (p < 0.02, Mann–Whitney test). The small number of responses to the IP-30 peptide in the non-HIV-infected population limited the significant difference to CMV-specific CD8^+^ T cells when individual comparisons between peptide-specific CD8^+^ T cells were done, but when grouped together, a significantly higher percentage of the CMV, EBV and flu peptide-specific CD8^+^ T cells expressed CD5 than the IP-30 peptide-specific CD8^+^ T cells (p < 0.02, Mann–Whitney test). These data indicate an increase in the percentage of CD5-negative peptide-specific CD8^+^ T cells in association with a low avidity TCR/peptide/MHC interaction in non-HIV-infected healthy control subjects.

**Table 1:** Selected peptides and corresponding avidities of TCR/HLA-A2/peptide interaction

**Peptide**	**Protein and location**	**Sequence**	**Avidity**
CMV	pp65 495-503	NLVPMVATV	1.0 nM
Flu	Matrix 55-63	GILGFVFTL	250 nM
EBV	BMLF1 280-288	GLCTLVAML	500 nM
IP-30	IP-30 -11 to -3	LLDVPTAAV	2.0 μM
Gag	p17 77-85	SLYNTVATL	50 nM
RT_1_	RT 33-41	ALVEICTEM	10 nM
RT_2_	RT 179-187	VIYQYMDDL	10 nM
PR	PR 76-84	LVGPTPVNI	0.1 nM

### Percentage of CMV, EBV, flu and IP-30 peptide-specific CD8^+^ T cells expressing CD5 in HIV infection

Data collected in the previous section allows us to test whether the overall reduction in percentage of CD8^+^ T cells expressing CD5 in HIV-infected individuals was reflected in non-HIV peptide-specific CD8^+^ T cells. To address this possibility, we next analyzed CD8^+^ T cells responding to non-HIV viral peptides and the IP-30 self-peptide in HIV-infected individuals. General characteristics of the HIV-infected study subjects are listed in Table 2. All subjects selected for testing were previously identified as expressing HLA-A2. For the 3 foreign peptides, medians of 98.4%, 97.6% and 96.7% of CMV, flu and EBV peptide-specific CD8^+^ T cells respectively from HIV-infected individuals expressed CD5, with no significant differences between peptides (Figure 3b). The percentage of CD8^+^ T cells reacting against the IP-30 self peptide that expressed CD5 (median = 88.4%) was significantly less than the percentages of CD8^+^ T cells reacting against the CMV, EBV and flu peptides either individually (p = 0.0004, p < 0.02 and p = 0.004 respectively, Mann–Whitney test) or grouped together (p = 0.0004, Mann–Whitney test). The percentages of CMV, EBV, flu and IP-30 peptide-specific CD8^+^ T cells expressing CD5 were not significantly different between the HIV-infected and healthy control groups, either when compared individually or grouped together as non-HIV viral peptide-specific CD8^+^ T cells. Therefore, HIV infection itself had no effect on CD8^+^ T cells specific for non-HIV viral peptides in terms of the percentage expressing CD5. As in the uninfected group, an increase in the percentage of CD5-negative T cells was associated with a low avidity TCR/peptide/MHC interaction involving a self peptide relative to those CD8^+^ T cells with higher avidity TCR/peptide/MHC interactions involving non-HIV viral peptides.

**Table 2:** General characteristics of HIV-infected study subjects

**ID**	**Age (years)**	**Sex**	**Duration of HIV infection (years)**	**CD4**^**+ **^**T cells/μL peripheral blood**	**Virus load**^**a**^	**Treatment**^**b**^
11	44	Female	>20	200	2.81	cART
12	40	Male	>20	518	<1.6	cART
18	37	Male	>20	729	1.97	cART
28	45	Male	>20	144	4.94	None
30	50	Male	>20	884	<1.6	cART
34	37	Female	>20	450	3.26	cART
35	52	Male	>25	882	1.6	cART
39	38	Female	>20	506	2.74	cART
43	49	Male	>20	450	4.15	None
45	42	Female	>20	456	<1.6	cART
62	38	Female	>20	720	<1.6	cART
65	54	Female	>20	306	<1.6	cART
71	38	Female	>20	860	<1.6	cART
78	41	Male	>20	150	2.73	cART
90	42	Female	>10	315	<1.6	cART
92	36	Female	>15	325	5.5	None
96	44	Female	>15	812	<1.6	cART
105	46	Male	>10	527	2.15	cART
115	44	Female	>10	704	<1.6	cART
117	45	Male	>15	322	<1.6	cART
125	51	Male	>10	420	<1.6	cART
126	50	Male	>10	754	<1.6	cART
132	37	Female	>15	30	5.53	None
134	44	Female	>20	780	<1.6	cART
151	54	Male	>10	360	<1.6	cART
154	47	Male	>20	442	<1.6	cART
155	41	Female	>5	500	<1.6	cART
179	44	Male	>5	984	<1.6	cART
181	39	Male	>20	104	4.79	None
193	43	Female	>5	420	<1.6	cART
196	54	Male	>5	273	<1.6	cART
197	59	Male	>5	616	<1.6	cART
201	52	Male	>5	434	<1.6	cART
204	42	Female	>20	437	<1.6	cART
213	55	Female	>5	561	<1.6	cART

^a^Virus load is expressed in log_10_ copies HIV RNA/ml plasma.

^b^Combination antiretroviral therapy (cART) represents various two or three class highly active antiretroviral drug regimens.

### Percentage of HIV peptide-specific CD8^+^ T cells expressing CD5

**Figure 4:**
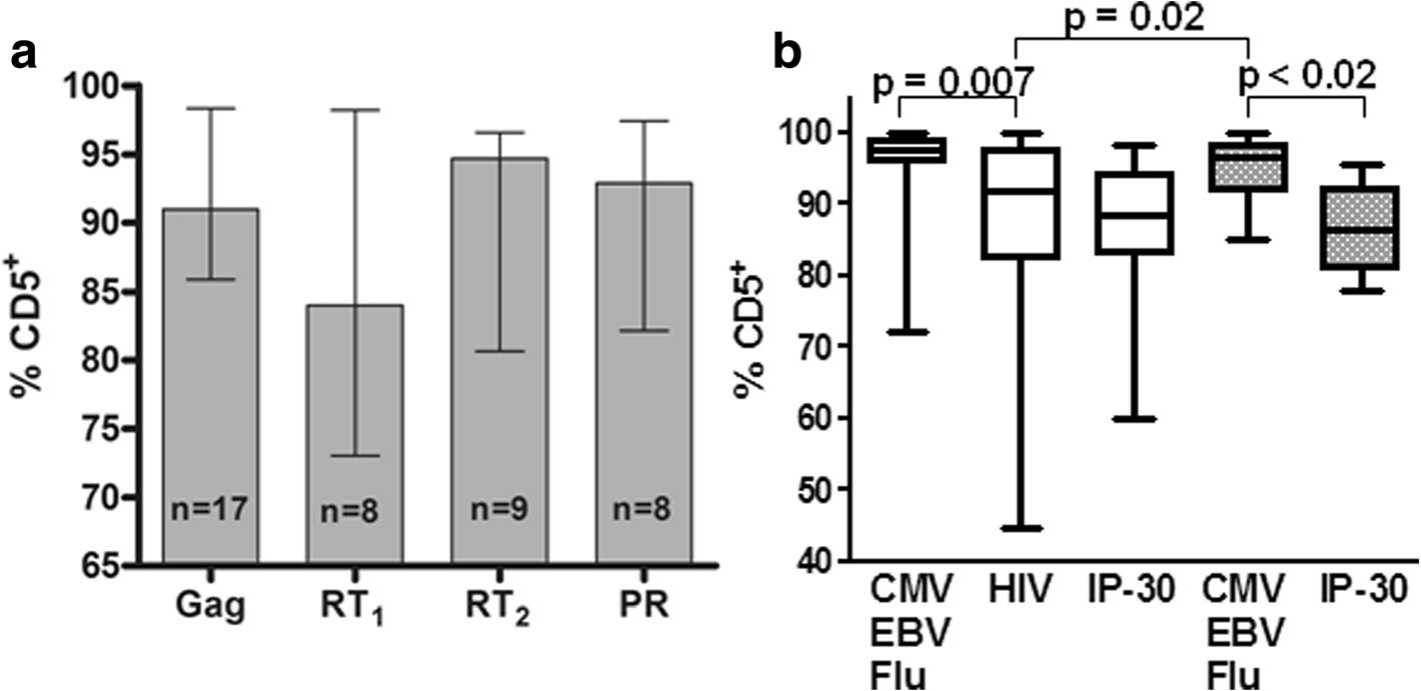
**The percentage of HIV peptide-specific CD8**^**+ **^**T cells expressing CD5 and comparison to other virus peptide and self-peptide-specific CD8**^**+ **^**T cells. (a)** Solid columns represent median values for each individual peptide (identified below the column) with error bars showing interquartile range and n within each column indicating the number of individuals with specific proliferation against each individual peptide. **(b)** Box and whisker plots showing median percentages of non-HIV viral peptide, HIV peptide and self peptide-specific CD8^+^ T cells expressing CD5 for healthy controls (grey boxes) and HIV-infected individuals (white boxes). Horizontal lines within the box represent the median, with boxes illustrating interquartile range and vertical error bars indicating overall range. Significant differences between medians are shown by p values above lines bracketing the relevant groups (Mann–Whitney test).

Next, we analyzed CD5 expression on CD8^+^ T cells responding to HIV peptides. The percentages expressing CD5 were generally lower than for CD8^+^ T cells responding to non-HIV peptides and similar to the percentages of CD8^+^ T cells reacting against the low avidity IP-30 self peptide (Figure 4a). There was no significant difference in the percentage of CD8^+^ T cells expressing CD5 between the 4 HIV peptides themselves, nor between any of the 4 HIV peptides, individually or in aggregate, and the IP-30 self peptide. Since there was no significant difference between CD5 expression levels of the individual HIV peptide-specific CD8^+^ T cell populations, we grouped them together for comparison with the non-HIV viral peptide and IP-30 peptide-specific CD8^+^ T cell populations of the HIV-infected and uninfected groups. A significantly lower percentage of HIV-specific CD8^+^ T cells expressed CD5 compared to the non-HIV viral peptide specific CD8^+^ T cells of HIV-infected (p = 0.0007, Mann–Whitney test) and healthy control (p = 0.02, Mann–Whitney test) groups (Figure 4b). The individual variability in the percentage of HIV peptide-responsive CD8^+^ T cells expressing CD5 was greater than for the non-HIV viral peptide-specific CD8^+^ T cells in either the HIV-infected or uninfected group (Figure 4b). These data indicate a broader range and selective reduction of the percentage of CD8^+^ T cells expressing CD5 within the HIV-specific CD8^+^ T cell population relative to the CD8^+^ T cells specific for other viral peptides in both the HIV-infected and healthy control groups. Features of the HIV-specific CD8^+^ T cells in terms of the range and median percentage expressing CD5 were similar to those of the self-peptide reactive CD8^+^ T cells.

### IP-30 self peptide reactivity

**Figure 5:**
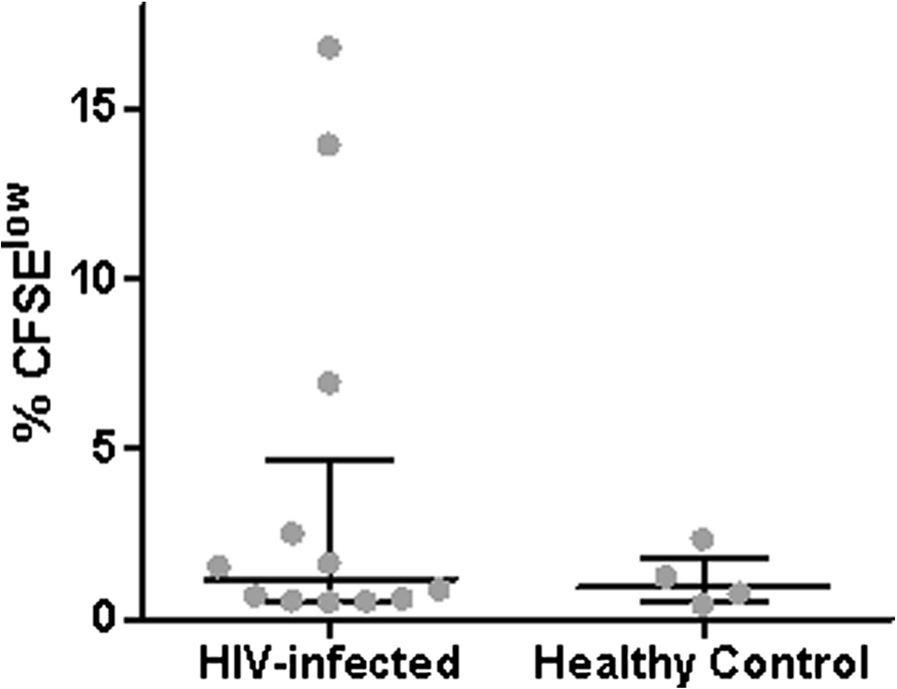
**Proliferative responses against IP-30 self peptide in healthy controls and HIV-infected individuals.** The percentage of CD8^+^ T cells proliferating in response to the IP-30 self peptide is shown for each of 4/24 healthy controls and 13/36 HIV-infected individuals with positive proliferative responses to the self peptide.

Since downregulation of CD5 on transgenic T cells is associated with manifestation of autoreactivity, we compared the frequency and levels of CD8^+^ T cell reactivity against the IP-30 self peptide in healthy control and HIV-infected study groups [14]. Proliferative CD8^+^ T cell responses against the IP-30 self peptide occurred in both the healthy control (4/24) and HIV-infected groups (13/36). There was a trend towards more frequent IP-30 self-peptide reactivity in HIV infection (p = 0.051, χ^2^ test) and several individuals in the HIV-infected group had notably strong proliferative responses to the IP-30 self peptide, but there was no significant difference in the median percentage of CFSE^low^ cells between IP-30 peptide responders in the HIV-infected versus healthy control groups (Figure 5).

## Discussion

The role of CD5 as a negative regulator of TCR signaling is well established and selective activation of variant CD5-negative T cell clones by tumour cells expressing low levels of HLA-class I/antigenic peptide complexes illustrates its biological relevance [15]. While modulation of CD5 under various conditions on transgenic T cells and selective expansion of CD5-negative T cells in HIV infection have been described, this is the first study comparing the percentage of CD5^+^ T cells from extended T cell repertoires responding against different peptides ranging in TCR avidity. The mean CD5-negative percentage of CD8^+^ T cells responding to three peptides from common viruses was low (6% or less) for both HIV-infected and control groups. There were no significant differences in the percentage of CD8^+^ T cells expressing CD5 between responses to the different peptides, despite TCR avidities ranging from 1.0-500 nM. There was also no significant difference between the HIV-infected and control groups for the three non-HIV viral peptides, either individually or in unison. However, in both the HIV-infected and control groups, the median percentage of CD5-negative CD8^+^ T cells responding to stimulation with a low TCR avidity (2.0 μM) self peptide was significantly higher than it was for the three peptides from common viruses. Although the four HIV peptides used for these studies had TCR avidities comparable to, or higher than the three non-HIV viral peptides, the median percentage of CD5-negative CD8^+^ T cells responding to stimulation with the HIV peptides was also significantly higher, and similar to the CD8^+^ T cell responses against the low avidity self peptide. This was not related to HIV infection itself as responses against non-HIV viral peptides were similar to uninfected controls in terms of the percentage of CD5-negative CD8^+^ T cells and the percentage of the general CD8^+^ T cell population of HIV-infected individuals expressing CD5 was significantly higher than in the CD8^+^ T cell responses against either the HIV peptides or self peptide. These data suggest that a general relationship between the percentage of CD5-negative CD8^+^ T cells responding against a peptide and TCR avidity may be apparent in responses against very low avidity self peptides, but not across a relatively broad range of avidities associated with peptides from three common viruses with low mutation rates compared to HIV. In contrast, the percentage of CD5-negative, HIV-specific CD8^+^ T cells responding against HIV peptides was inconsistent with their TCR avidities relative to the other viral peptides and similar to that seen in responses against the low avidity self peptide.

While CD8^+^ T cell responses to only a few peptides and viruses in the context of just one HLA-restriction molecule (HLA-A2) were studied, we speculate that this apparent inconsistency in CD5 expression levels could reflect the diverse in vivo representation of HIV peptides within viral quasispecies. The sequence of wild-type or reference peptides used for in vitro stimulation is actually based on a consensus drawn from multiple variants. Sequence variation generated in vivo probably goes well beyond that observed in the viruses that successfully achieve some level of prominence. Thus, for each HIV peptide epitope defined by a reported consensus sequence, the CD8^+^ T cells of an infected individual are most likely exposed to multiple variants in vivo, many of which will continue to be presented by the relevant HLA class I molecule. Rather than a single TCR/MHC peptide interaction with a unique associated avidity, this would produce a spectrum of interactions and avidities imposing different constraints on CD5 expression levels compatible with T cell activation. In vivo adaptation to the many variants producing a generally lower and individually more variable percentage of CD5-expressing CD8^+^ T cells responding to the wild-type peptide in vitro is one possible explanation for the results observed in our study.

Such in vivo adaptation in the percentage of responding cells expressing CD5 could have positive and negative implications. Cells lacking CD5 would be selectively activated and mediate broader recognition of variant peptides arising in vivo than their CD5 expressing counterparts. However, transgenic TCR studies suggest the CD5-negative CD8^+^ T cells would also be more likely to mediate autoimmunity by reacting against self-peptides with low avidity TCR interactions and to skew the T cell repertoire through a selective advantage for homeostatic proliferation [12,13,16]. This feature might underlie the more substantial fractional expansion of CD5-negative CD8^+^ T cells reported in HIV-infected individuals in the study carried out prior to the introduction of HAART [18]. In secondary haemophagocytic lymphohistiocytosis, often driven by EBV infection, there is a rapid and profound oligoclonal expansion of activated CD5-negative T cells expressing EBV RNA [22,23]. The oligoclonality of the CD5-negative cells suggests they may be involved in the immune response against EBV and that down-regulation of CD5 facilitates their rapid expansion. This may facilitate rapid generation of a massive oligoclonal response that effectively suppresses replication of a relatively stable DNA virus like EBV. In this case, down-regulation of CD5 could be an effective antiviral adaptation to amplify the immune response, with only a transient effect on the CD8^+^ T cell population due to heightened sensitivity of CD5-negative CD8^+^ T cells to apoptosis [24]. In this scenario, down-regulation of CD5 could also be advantageous for tumour infiltrating lymphocytes. However, the association between EBV infection and CD5 down-regulation could actually reflect direct infection of the CD8^+^ T cells with EBV rather than the immune response against EBV [22,23].

Adaptive regulation of CD5 expression on T cells may amplify the immune response in certain settings and conversely, activate Treg cells in others [25]. The Ets family E47 transcription factor and IL-7 negatively regulate CD5 expression through poorly understood mechanisms [12,26,27]. Better understanding of the impact, molecular basis and dynamics of CD5 regulation on different T cell subsets could reveal new methods to modulate immunity in pathological settings.

## Conclusions

There is a general relationship between a reduced percentage of responding CD8^+^ T cells expressing CD5 and reactivity against the common HLA-A2-restricted low avidity self peptide used in this study. While this relationship was not seen across a set of common viral peptides spanning a 500 nM range of avidity, we also observed reduced CD5 expression on CD8^+^ T cells reactive against four HIV peptides similar to that seen with CD8^+^ T cells responding against the low avidity self peptide. Since HIV mutates extensively, one possible explanation is that down-regulation of CD5 on HIV-specific CD8^+^ T cells represents in vivo adaptation to HIV variant peptides with lower avidities than the reference peptides used to assess reactivity. This adaptation would have potential for positive consequences in terms of functional recognition of a broader set of viral variants, but also for negative consequences related to autoreactivity and narrowing of the T cell repertoire.

## Methods

### Subjects

Healthy controls were recruited from laboratory and hospital personnel and HIV-1-infected subjects recruited through the Newfoundland and Labrador Provincial HIV Clinic in St. John’s, Newfoundland. Ethical approval for this study was granted by the Newfoundland and Labrador Health Ethics Research Authority and all participants provided written informed consent for blood collection and immunological studies. Peripheral blood was obtained by forearm venipuncture into vacutainers with acid citrate dextrose anticoagulant (Becton Dickinson) and peripheral blood mononuclear cells (PBMC) were isolated by ficoll-hypaque plus (GE Healthcare) density gradient centrifugation. Isolated PBMC were washed and resuspended in lymphocyte medium consisting of Roswell Park Memorial Institute (RPMI) 1640, 10% fetal calf serum (FCS), 10 mM HEPES, 2 mM L-glutamine, 100 IU/mL penicillin, 100 μg/mL streptomycin and 2 × 10^-5^ M 2-mercaptoethanol (all from Invitrogen). The cells were then counted and used directly for human leukocyte histocompatibility-linked antigen (HLA) typing with the remaining cells resuspended at 1.0 × 10^7^/mL in medium with 20% FCS and 10% dimethylsulfoxide (Sigma) for preservation in liquid N_2_ until use. All subjects were typed for class I A and B HLA antigens by serologic assay as previously described and those expressing HLA-A2 chosen for further study [28].

### Peptide selection

A selection of peptides presented to CD8^+^ T cells by HLA-A2 were used in this study, including immunodominant peptides from common viruses to which most individuals are exposed: cytomegalovirus (CMV) pp65 495–503 (NLVPMVATV); Epstein-Barr virus (EBV) BMLF1 280–288 (GLCTLVAML); influenza (flu) A Matrix 55–63 (GILGFVFTL), interferon inducible protein (IP)-30 -11 to -3 (LLDVPTAAV) self-peptide; HIV-1 peptides commonly recognized by subjects in our cohort; Gag P17 77–85 (SLYNTVATL); Pol reverse transcriptase (RT_1_) 33–41 (ALVEICTEM); Pol RT_2_ 179–187 (VIYQYMDDL); Pol protease (PR) 76–84 (LVGPTPVNI). The TCR avidities of these peptides reported in the literature span a 3 log range, which we confirmed with our own reactive samples. Peptides were synthesized and purified to > 95% purity by EZBiolab.

### ELISPOT assays

Twenty-four HLA-A2 healthy control individuals were tested by interferon-gamma (IFN-γ) ELISPOT for CD8^+^ T cells specific for the CMV, flu, EBV and IP-30 self peptides. Multiscreen microtitre plates (Millipore) were coated overnight at 4°C with 100 μL/well of 7.5 μg/ml anti-IFN-γ monoclonal antibody (mAb) 1-D1K (MAbtech) and then washed 3 times with phosphate-buffered saline (PBS). Duplicate wells were incubated overnight with 200 μL total volume lymphocyte medium containing 4 μg/mL of either CMV, EBV, flu or IP-30 peptide and 1×10^5^ PBMC from the subject being tested. Cells recovered from liquid N_2_ were cultured overnight before counting to ensure viability. Medium alone served as negative control and 5 μg/mL phytohemagluttinin (PHA, MP Biomedicals) as positive control. After overnight (16 hr) incubation, PBMC were removed from the wells, the plates washed 6 times and 100 μL of 1 μg/mL biotinylated anti-IFN-γ mAb 7-B6-1 (MAbtech) added for 2 h. Plates were washed 6 more times and 100 μL/well of a 1/1000 dilution of streptavidin-alkaline phosphatase (AP) (MAbtech) added for 1 h. Plates were washed 6 more times and 100 μL/well chromogenic AP substrate (BioRad) added. After spots developed (~30 min), plates were washed with tap water to stop reactions and then air-dried. Spots corresponding to cytokine-producing T cells were counted on a Cellular Technology Limited Immunoscan reader. Subjects were considered to have detectable memory CD8^+^ T cells against the relevant peptides when the number of spot forming cells (sfc) was > 200/10^6^ PBMC after subtraction of background spots in negative control wells. Thirty-six HIV-infected subjects expressing HLA-A2 were tested by ELISPOT as above for CD8^+^ T cell reactivity with the non-HIV peptides and with the 4 common HIV-1 peptides also presented by HLA-A2 and listed above.

### Avidity determination

Avidity levels reported in the literature for TCR interactions with the relevant peptide/HLA-A2 complexes were confirmed as previously described by serial dilution of the peptides in cell-mediated cytotoxicity assays [21]. Briefly, cytotoxic T cells (CTL) specific for the peptide were generated by incubating 5 × 10^6^ PBMC from a reactive subject in a small volume of medium for 1 hr with 100 μM peptide and then culturing the cells at 2.5 × 10^6^/mL in medium supplemented with 25 ng/mL recombinant interleukin-7 (R&D Systems). After 3 days, interleukin-2 (Roche) was added to 10 U/mL and the cells expanded for a further 7 days. The effector cells were first tested against autologous or HLA-A2 matched ^51^Cr-labelled BLCL pulsed for 1 hr with 10 μM peptide as in reference [20] to determine an appropriate effector-to-target (E:T) ratio and then were tested against target cells pulsed with a serial dilutions of the test peptide beginning at 1 μM and progressing through 5 fold dilutions to < 0.02 nM. The avidity of the TCR/peptide-Class I HLA complex interaction was determined by graphing percent specific cytotoxicity against peptide concentration and estimating the concentration of peptide at which specific cytotoxicity fell to 50% of maximum cytotoxicity observed.

### Proliferation assays

To evaluate CD5 expression on peptide-specific CD8^+^ T cells, 2×10^6^ PBMC were labeled with 1 μM carboxyfluorescein succinimidyl ester (CFSE, Invitrogen) as per the manufacturer’s instructions, washed, and incubated in a small volume with either lymphocyte medium alone or 100 μM peptide of interest for 1 hour, then resuspended and cultured in 1 mL lymphocyte medium (with human serum substituted for FCS) for 7 days. Peptide-specific T cells were identified by CFSE fluorescence intensity dilution using a Becton Dickinson FacsScan flow cytometer. CD8^+^CD3^+^ cells were identified using phycoerythrin (PE)-conjugated anti-CD3 and peridinyl chlorophyll protein (PerCP)-conjugated anti-CD8 (clones UCHT1 and HIT8a respectively, BioLegend). The fraction of proliferating cells expressing CD5 was estimated by co-staining the cells with allophycocyanin-conjugated anti-CD5 (clone L17F12, eBioscience), gating on CD8^+^CD3^+^ cells and plotting CD5 versus CFSE intensity. Positive peptide-specific proliferation was defined as at least twice the percentage of proliferating cells compared to the control culture. If background proliferation in the absence of stimulation was > 2.5%, results were excluded. The baseline fraction of CD8^+^ T cells expressing CD5 was measured by flow cytometry with fresh PBMC using the same antibodies.

### Statistical analysis

Data analysis and graphical representation was done with GraphPad Prism software, v4.03. Normality of data distribution was assessed by Kolmogorov-Smirnoff and D’Agostino Pearson tests, but medians with interquartile range were shown since not all data sets in any of the comparisons had normal distributions. Differences in the median percentages of different CD8^+^ T cell populations expressing CD5 were assessed with the two tailed non-parametric Mann–Whitney test.

## Competing interests

The authors declare that they have no competing interests.

## Authors’ contributions

SJP carried out the research described in this manuscript, analyzed the data and contributed to drafting the manuscript. MEG processed samples, carried out the HLA typing and collected some of the data presented in this manuscript. MDG conceived and designed the study, directed the research, modified the figures for presentation and finalized the manuscript for submission. All authors read and approved the final manuscript.
